# Biaxial seismic response of base-column connections in sub-standard steel buildings: dataset

**DOI:** 10.12688/openreseurope.17563.2

**Published:** 2024-11-27

**Authors:** Nikolaos Stathas, Luigi Di Sarno, Jingren Wu, Fabio Freddi, Mario D’Aniello, Stathis Bousias, Raffaele Landolfo, Esra Mete Güneyisi, Lambros Papagiannis

**Affiliations:** 1 Civil Engineering, University of Patras, Patras, Peloponnisos Dytiki Ellada ke Ionio, 26504, Greece; 2 Civil and Environmental Engineering, University of Liverpool, Liverpool, England, L69 7ZX, UK; 3 Structures for Engineering and Architecture, University of Naples Federico II, Naples, Campania, 80125, Italy; 4 Institute of Risk and Disaster Reduction, University College London, London, England, WC1E 6BT, UK; 5 Civil, Environmental and Geomatic Engineering, University College London, London, England, WC1E 6BT, UK; 6 Civil Engineering, Gaziantep University, Gaziantep, Gaziantep, 27310, Turkey

**Keywords:** Column-base steel connections, sub-standardard buildings, steel frame connections, pseudodynamic method, biaxial testing

## Abstract

Existing steel frames not complying with modern seismic codes are often vulnerable to earthquakes due to inadequate seismic detailing. These types of framed structures typically feature semi-rigid and partial strength column-base connections; the behaviour of such connections may significantly affect their seismic performance. However, current code provisions offer limited guidance for the assessment and retrofit of column-base connections To fill the knowledge gap, the H2020 EU-funded Earthquake Assessment of Base-Column Connections in Existing Steel Frames project experimentally investigated, the response of exposed column-base plate connections. Bi-directional Pseudo-Dynamic tests were carried out at the Structures Laboratory of the University of Patras within the framework of "Engineering Research Infrastructures for European Synergies - ERIES" project. The case-study steel frame featured two types of column-base plate connections, *i.e.,* stiffened and unstiffened, representing respectively the base connections of an external moment-resisting frame and an internal gravity frame. The experimental programme comprised free vibration tests to identify the modal properties of the sample steel frame. A set of quasi-static cyclic tests and pseudo-dynamic tests were then carried out to investigate the performance of the steel frame under bi-directional earthquake sequences. The response of each component constituting the column-base plate connections was monitored during the tests to fully capture the behaviour of the connections. Such experimental results allow model calibration and further parametric investigation on column base plate connections.

## Plain language summary

The international building stock comprises structures built decades ago, on the basis of past knowledge and before the advent of modern seismic codes. Some critical connections of such steel structures (e.g. the plates welded at the bottom of columns to secure their connection to the foundation) may be particularly vulnerable to earthquakes, especially when they strike the structure concurrently from two transverse directions: the plates may deflect and uplift, accumulating deformations which may endanger their integrity. Since earthquakes do not occur only once in the life span of a structure, these structural parts are subjected to cumulative damage placing under question their capacity to sustain future earthquakes.

Under support provided by the European Commission, the project aims at studying this phenomenon by constructing and testing in a specialized European research laboratory a steel building - representative of this class of structures - at full scale. Employing an innovative experimental technique to impose repetitive biaxial seismic excitation on the specimen and through a large number of sensors, valuable data for the local and global response of the frame was possible. The data are made openly available for re-use by the scientific and professional engineering community.

## Introduction

The poor performance of column-base connections observed in the aftermath of several seismic events highlighted the significant need to advance current design and assessment strategies and to gain a more comprehensive and in-depth understanding of their behaviour ( *e.g.,* Clifton *et al.,* 2011; Di Sarno *et al.,* 2018; Gutiérrez-Urzúa *et al.,* 2021; Mahin, 1998; Okazaki *et al.,* 2013; among many others). Column-base connections typically need to transfer concurrently axial and shear forces, as well as bending moments from the superstructure to the foundation(s). Previous research has investigated the behaviour of different layouts of column-base connections, such as exposed base plate connections ( *e.g.,* Fasaee *et al.,* 2018; Latour & Rizzano, 2013a; You & Lee, 2020) and embedded connections ( *e.g.,* Di Sarno *et al.,* 2007; Grilli *et al.,* 2017; Inamasu *et al.,* 2021). Exposed base plate connections have conventionally been used in many European countries for low- to medium-rise steel frames. These connections consist of steel plates welded onto the column base and anchored to the foundation block through steel bolted bars. Stiffeners are also frequently used to enhance the rigidity of connections.

Current European codes offer limited guidance on the assessment and retrofit of column base connections. For example, the framework implemented in Eurocode 8-Part 3 ( CEN, 2005b) for assessing existing steel buildings primarily accounts for beam-to-column connections and does not provide explicit guidance for column-bases. Besides, the component method adopted in the Eurocode 3-Part 1.8 ( CEN, 2005a) predicts rotational stiffness and moment capacity of base-column connections. However, it does not allow the definition of the whole moment-rotation relationship, which requires a proper definition of the ultimate deformation capacity ( Latour & Rizzano, 2013b; Latour *et al.,* 2014).

Significant research effort has been dedicated recently to steel column-base connections. However, from one side, there has been a limited amount of experimental work; on the other side, most of the tests focused on uniaxial bending ( *e.g.,* Borzouie *et al.,* 2015; Borzouie *et al.,* 2016; Kanvinde *et al.,* 2012; Latour *et al.,* 2014; Lee *et al.,* 2008a; Lee *et al.,* 2008b; Zhou *et al.,* 2004) and only a limited number of studies investigating the influence of biaxial bending ( *e.g.,* Cloete & Roth, 2021; Seco *et al.,* 2021). Nevertheless, the performance of column-base plate connections has rarely been investigated through testing full-scale steel frames under bi-directional loading. There is also limited investigation into the impact of cumulative damage on such column-base connections resulting from repeated earthquakes.

To fill the knowledge gap, the H2020 EU-funded Earthquake Assessment of Base-Column Connections in Existing Steel Frames (HITBASE) project experimentally investigated the response of exposed column-base plate connections. Bi-directional Pseudo-Dynamic tests were carried out at the Structures Laboratory (STRULAB) of the University of Patras within the framework of Engineering Research Infrastructures for European Synergies (ERIES). The tests focused on a large-scale specimen extracted from a non-seismically designed steel frame. The specimen included two column-base plate connection typologies, *i.e.,* stiffened and unstiffened, representing the base connections of external moment-resisting and internal gravity frames.

To facilitate data availability to the research community, the data which resulted from the testing campaign is stored to the public repository Zenodo and made openly available. This paper provides rich metadata to be employed in understanding and re-using the data provided by describing the experimental campaign and providing information on properties of the test specimens, construction, instrumentation, testing procedures, and the test matrix - some brief observations during the tests are included as well (more detailed description of specimen response will be covered in subsequent publications by the research team).

## Description of the specimen

**Figure 1.**
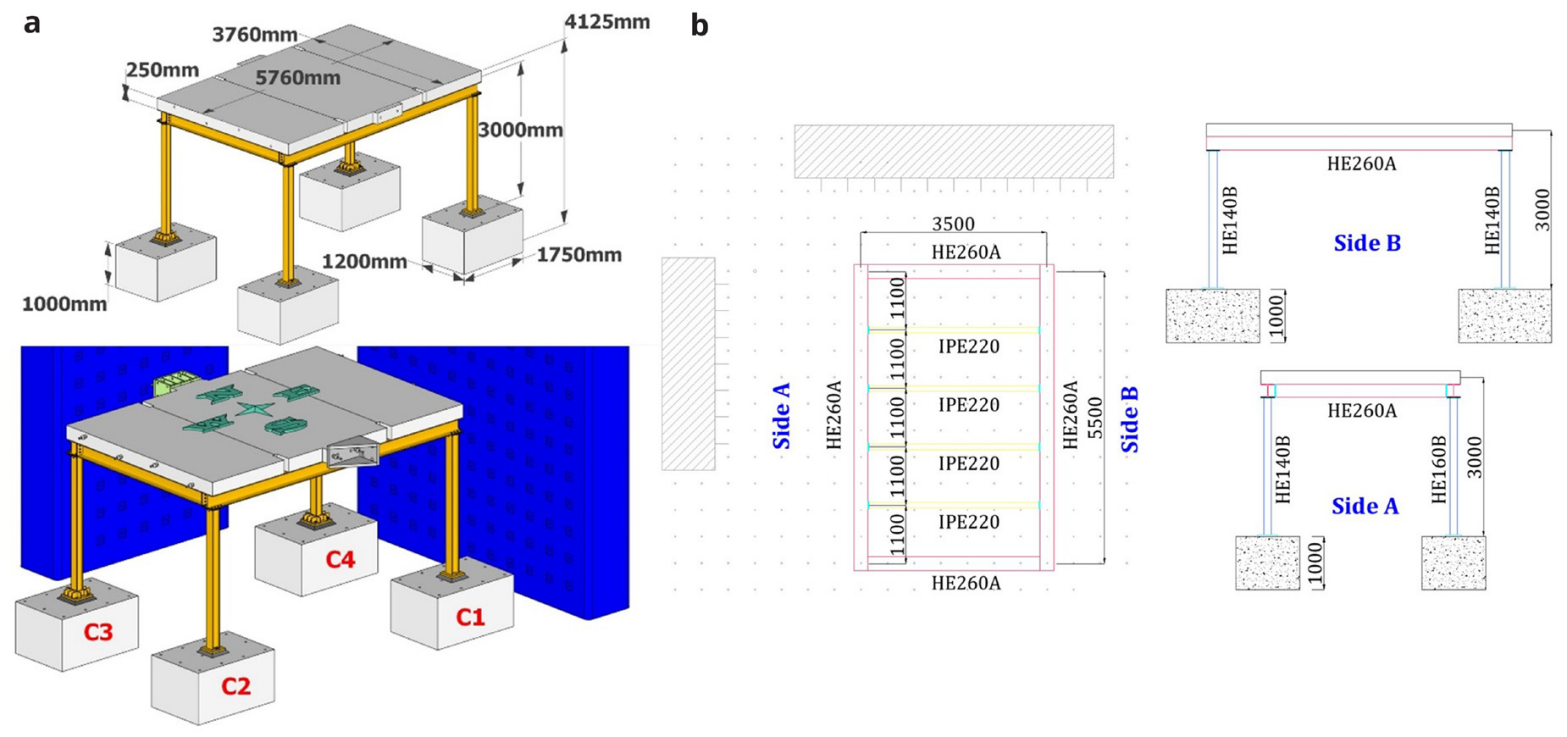
Specimen geometry: ( **a**) general view and column-naming; ( **b**) plan-view and elevation (unit: mm).

The test specimen is a full-scale, single-story, one-by-one bay steel frame representing a sub-structure of a non-seismically designed structure. The 5.75 m-long by 3.65 m-wide specimen features a height of 3 m to the mid-depth of a 0.25 m-thick reinforced concrete slab ( Figure 1). The two columns on the South side of the structure (columns C1 and C2 in Figure 1a) have a HE160 section, while their counterparts on the North side (columns C3 and C4 in Figure 1a) have a HE140 section, thus resulting in a torsionally unbalanced structure. The perimeter beams are characterised by HE260 sections, while the four secondary transverse beams placed in the N-S direction at spacings of 1.10 m are made of IPE 220 sections.

All columns rest reinforced concrete footings with dimensions of 1 m × 1.75 m × 1.20 m (see Figure 1) made of C20/25 class concrete. The reinforced concrete slab, also made of C20/25 concrete, is connected to both perimeter and intermediate beams through 20 mm-in-diameter dowels spaced at 200 mm. All steel sections are built of S355 grade.

**Figure 2.**
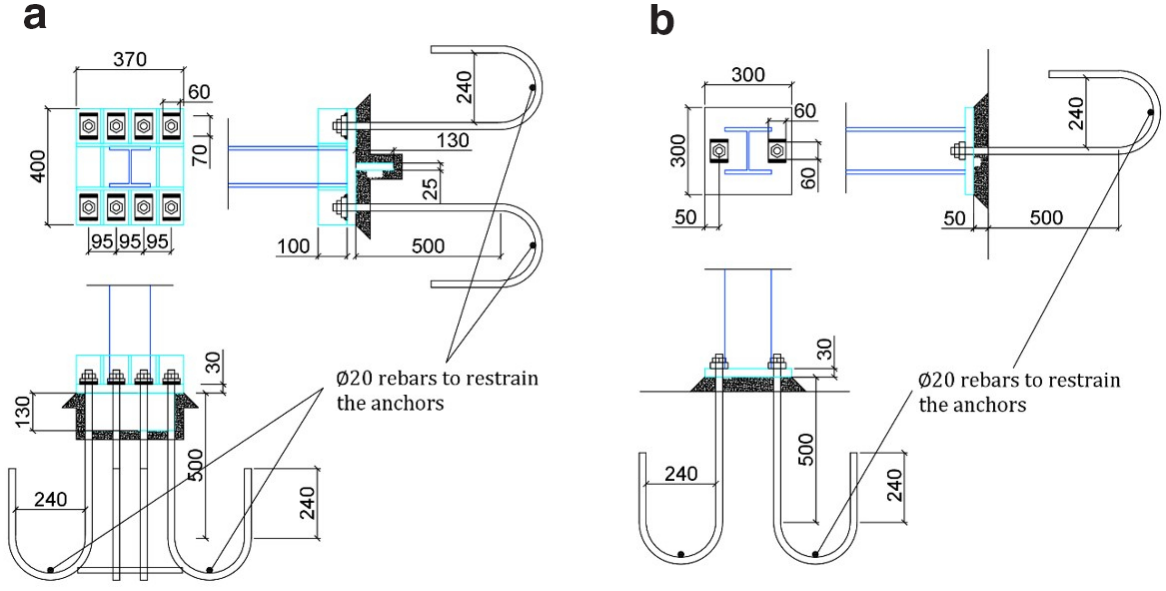
Details of columns' base connection to foundation, for ( **a**) columns C3–C4 and ( **b**) columns C1 -C2 (unit: mm).

Different column-foundation connection details are used for columns on the North and South sides of the structure. Columns C1 and C2 ( *i.e.,* HE160 section) are welded via full-penetration welds to a 300-mm square 30 mm-thick bottom steel plate, which is anchored on the foundation via two Μ24 bars ( Figure 2a). No stiffening plates are provided at the base of these two columns. On the other side, columns C3 and C4 ( *i.e.,* HE140 section) bear a stiffer connection to the foundation ( Figure 2b) via a number of stiffening plates above the 370 × 400 mm base plate (30 mm-thick), where a 310 × 130 mm 25 mm-thick plate is welded underneath it and protrudes into a pocket in the foundation. Eight Μ24 bars of grade S355 steel are employed to provide anchorage to the RC foundation, which are post-tensioned to 150 kN. In all cases, a 50 mm-thick pad of high strength mortar is placed between each column base-plate and foundation, while for C3-C4 columns, the mortar is forced to also fill the pocket in the foundation for properly encasing the plates welded underneath the relevant base plates.

**Figure 3.**
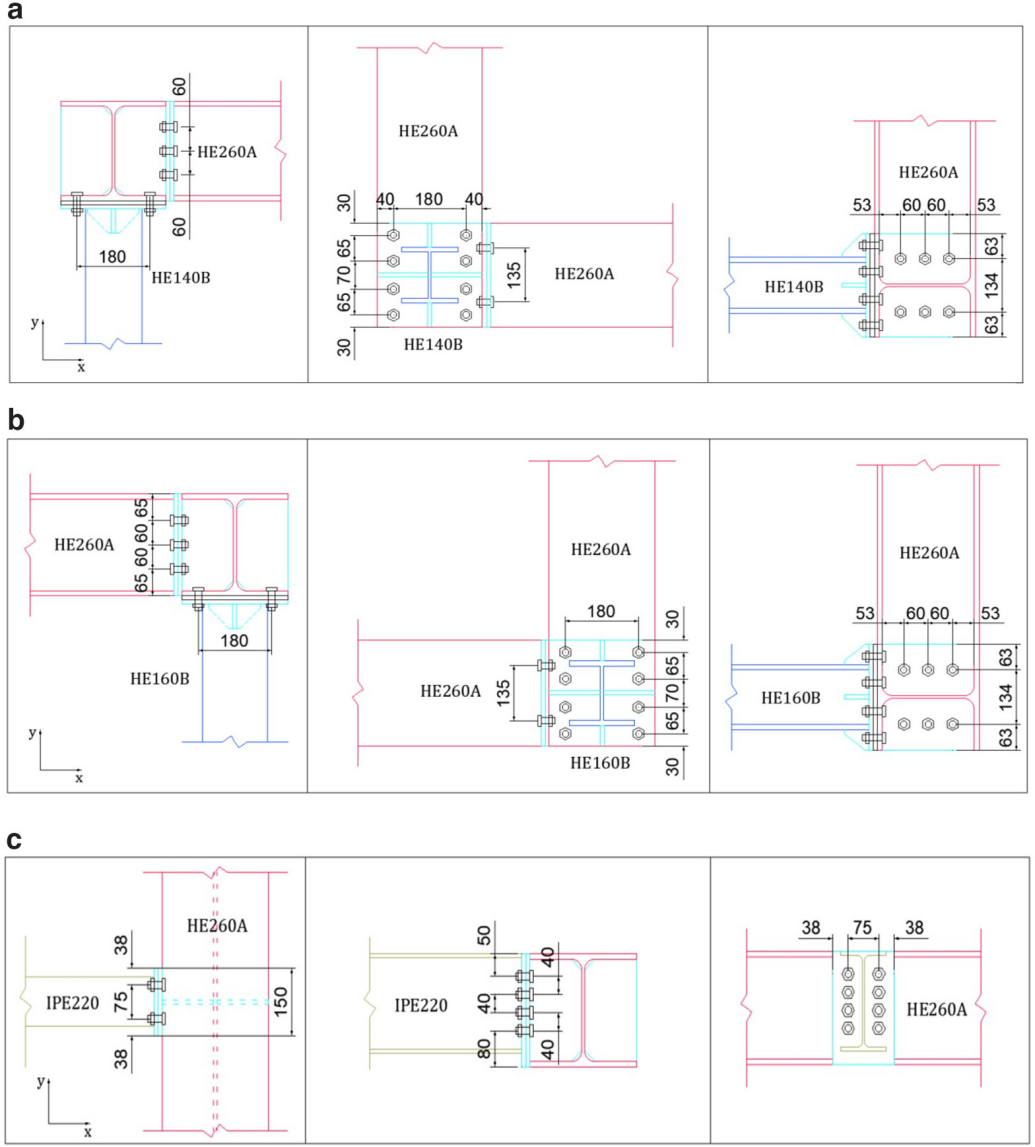
Details of connections: ( **a**) HE140-column-beam (North-side), ( **b**) HE160-column-beam (South-side), and ( **c**) secondary-to-perimeter beam (unit: mm).

Moreover, the details of the beam-to-column, beam-to-beam and perimeter-to-secondary beam connections are depicted in Figure 3. For all these connections, 10.9 grade M16 bolts which are post-tensioned at 120 kN are employed.

The mechanical properties of the materials, as obtained from relevant material characterisation tests are summarised in Table 1. The mean compressive strength of the C20/25 concrete used for slab and foundation was determined over six samples, which are 32.3 and 34.2 MPa respectively. Moreover, the yield and ultimate strength of the S355 steel were also determined for each steel profile used by the specimen. The yield strength was measured to be between 370 and 431 MPa, while the ultimate strength was between 487 and 560 MPa. In addition, the mean compressive strength of the mortar was 47.1 MPa, measured on 50-mm cube samples.

**Table 1.** Mechanical properties of materials (units: MPa).

	Concrete (C20/25)		Steel (S355)				Mortar
	Slab	Foundation	HE 260A	HEB 140	HEB 160	IPE220	
Mean strength	32.3	34.2					47.1
Yield strength			431	370	431	391	
Ultimate strength			549	505	560	487	

## Test setup and instrumentation

**Figure 4.**
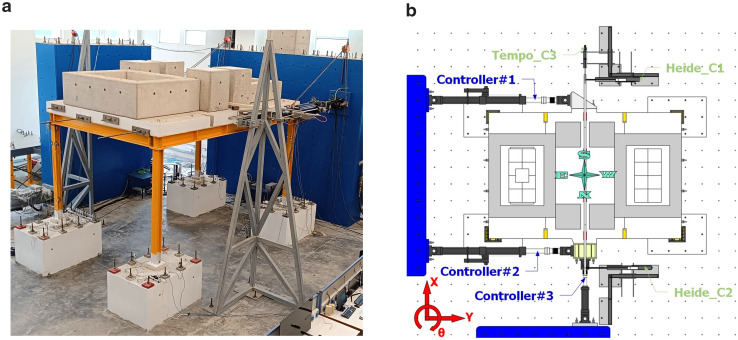
( **a**) Photo of the setup and ( **b**) control equipment and naming scheme.

The test setup is shown in Figure 4. Additional masses ( *i.e.,* concrete blocks) of 15.76 tons were placed on the slab to simulate the mass of non-structural components and other loads. With the size of the slab exceeding crane capacity and the perimeter beams being permanently connected to the slab via dowels, dismantling the slab-plus-beams ensemble after testing would have been problematic. This issue was solved by pouring the slab in three different phases, not allowing for reinforcement to cross the interface sections and by finally post-tensioning the three pieces to guarantee monolithicity.

The test aimed to investigate the response of the structure along both directions. Employing pseudo-dynamic test of an asymmetric 3D-frames, the testing method requires the use of sufficient number of actuators to provide the deformation pattern sought, *i.e.,* translational displacements along the main axes, plus rotation about the vertical one. The loading scheme providing the required kinematics comprises a pair of actuators acting in tandem along the long direction of the frame and a third one acting in the transverse direction. The former controls frame displacement along the longitudinal dimension as well as its rotation, whilst the latter contributes to the control of frame displacement along the transverse direction.

The test process relies on the step-wise Pseudo-Dynamic method and involves a numerical and an experimental (physical) part: the former focuses on the step-by-step solution of the equation of motion of the system under a selected pair of seismic signals in two transverse directions. The resulting deformation vector (defined at the centre of mass of the system) comprises two transverse displacements and a rotation and, after being projected along the instantaneous directions of the longitudinal axis of each actuator, is applied to the structure - exact application of the displacement command signals is monitored via external, high resolution (2 μm) optical transducers ( Figure 4b). The resulting reaction forces measured by the load cell on each actuator are transformed backwards to force components along the axes of the frame and sent to the numerical part to advance to the next load step.

**Figure 5.**
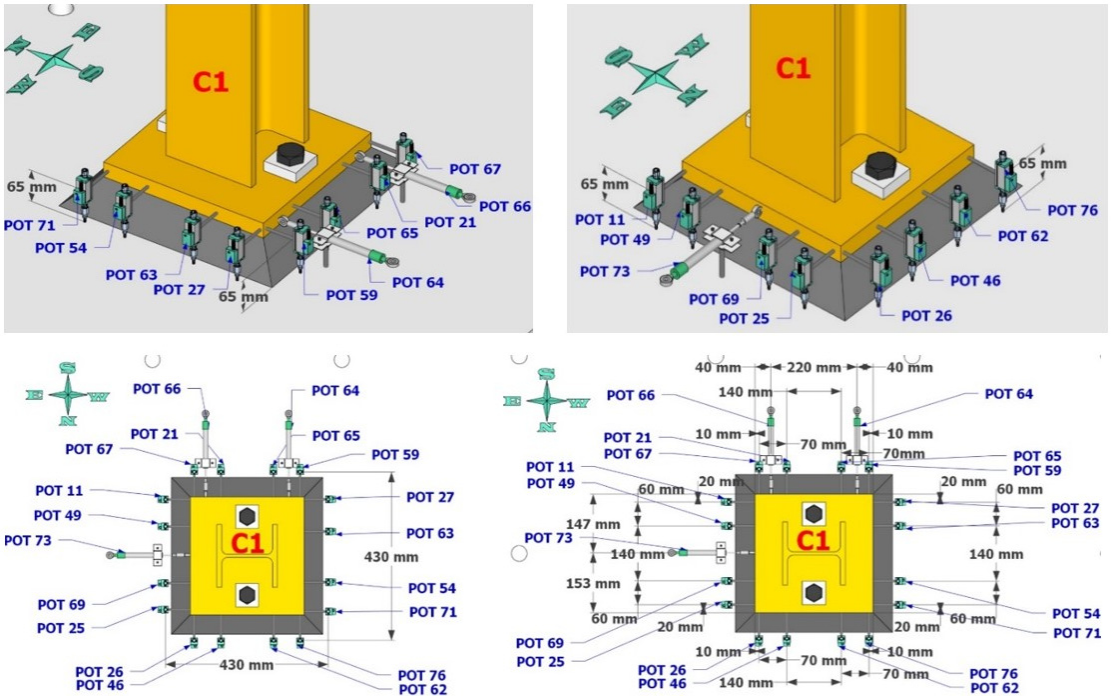
Potentiometers recording lateral and vertical deformations at the base of column C1.

Dense specimen instrumentation was employed on two diagonally opposite columns (C1 and C3 in Figure 1a). Response parameters (strain, deformation, inclination) at several points/sections were monitored - the type/position of the sensors is shown in Figure 5– Figure 10.

**Figure 6.**
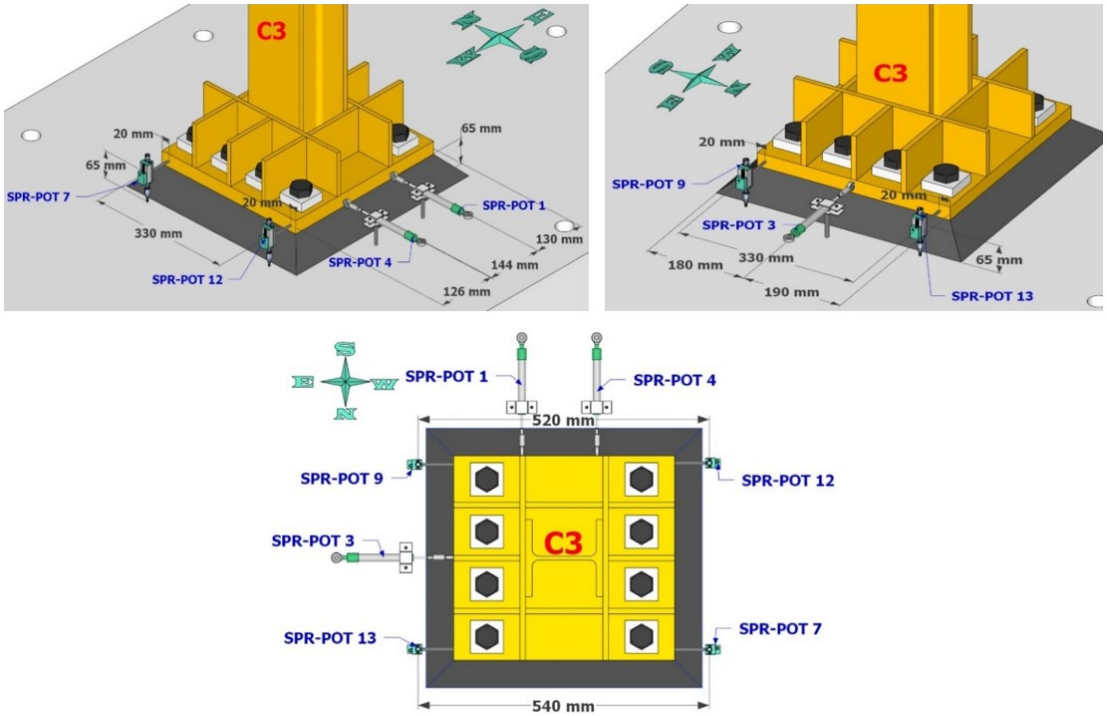
Potentiometers recording lateral and vertical deformations at the base of column C3.

**Figure 7.**
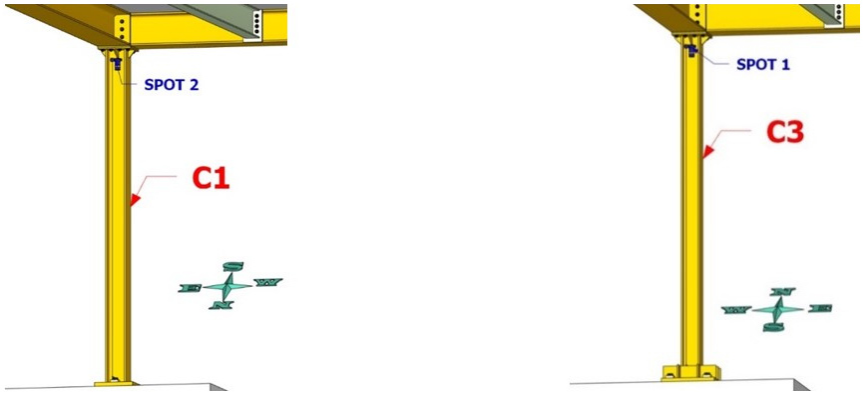
Position of string potentiometers measuring axial deformation along columns C1, C3.

**Figure 8.**
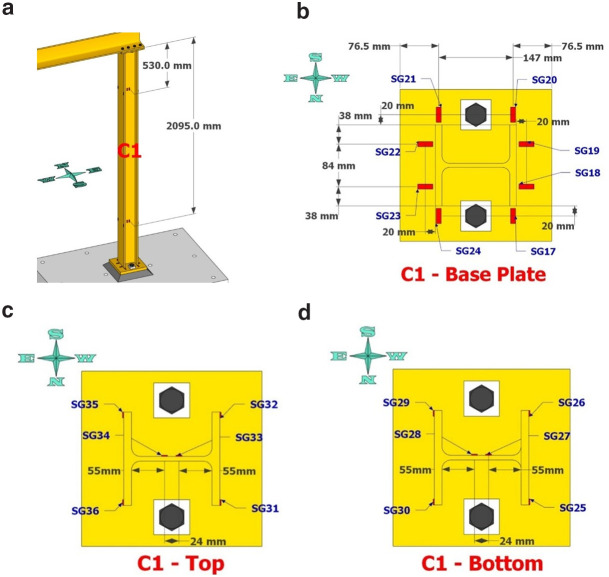
Column C1 strain-gage instrumentation: ( **a**) at 530- and 2095mm from column top; ( **b**) gages on base plate; ( **c**) gages as placed at section 530mm from top; ( **d**) gages at section 2095mm from top.

**Figure 9.**
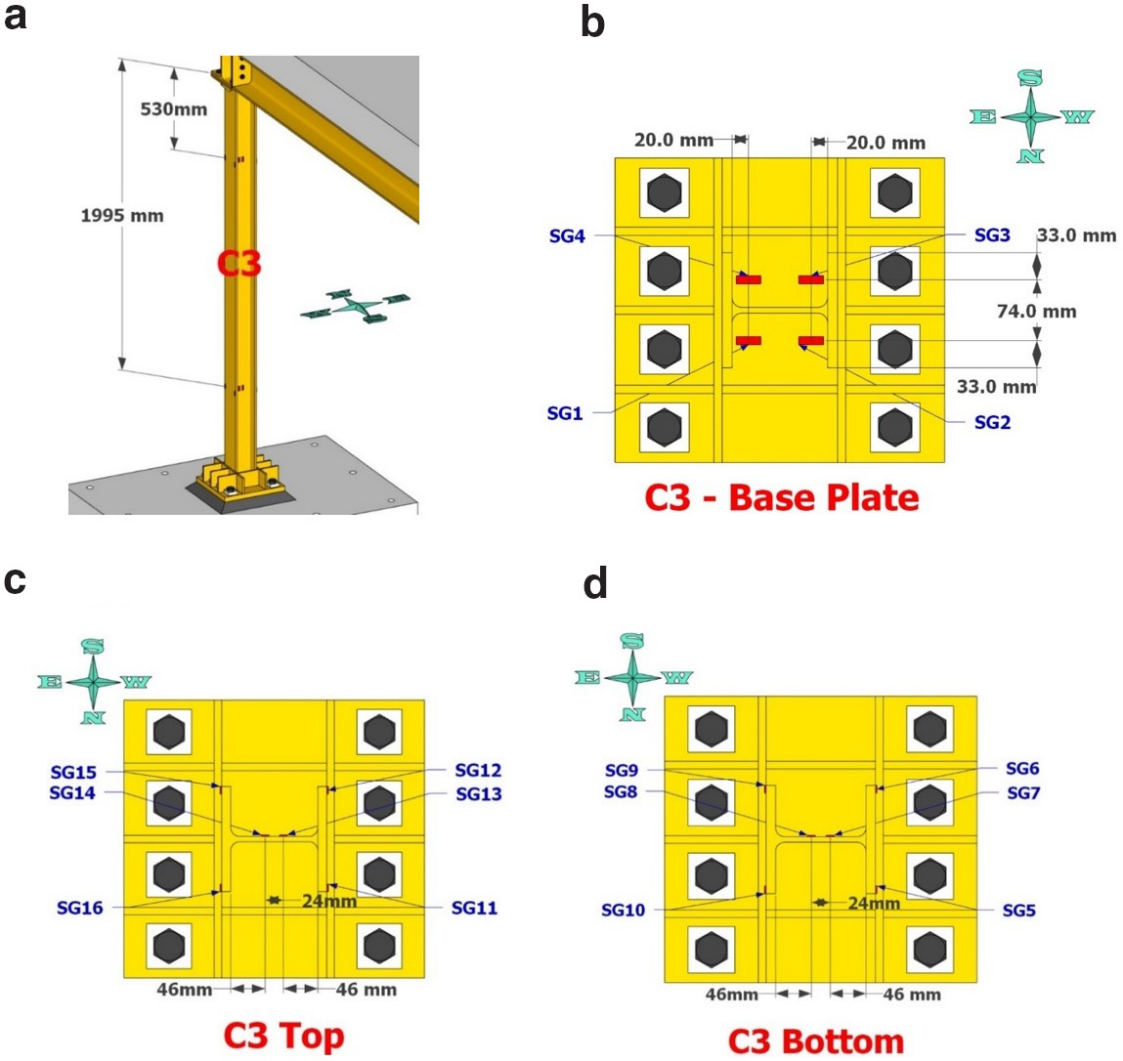
Column C3 strain-gage instrumentation positioning: ( **a**) along column height; ( **b**) on base plate; ( **c**) at 530mm from top; ( **d**) at 1995mm from top.

**Figure 10.**
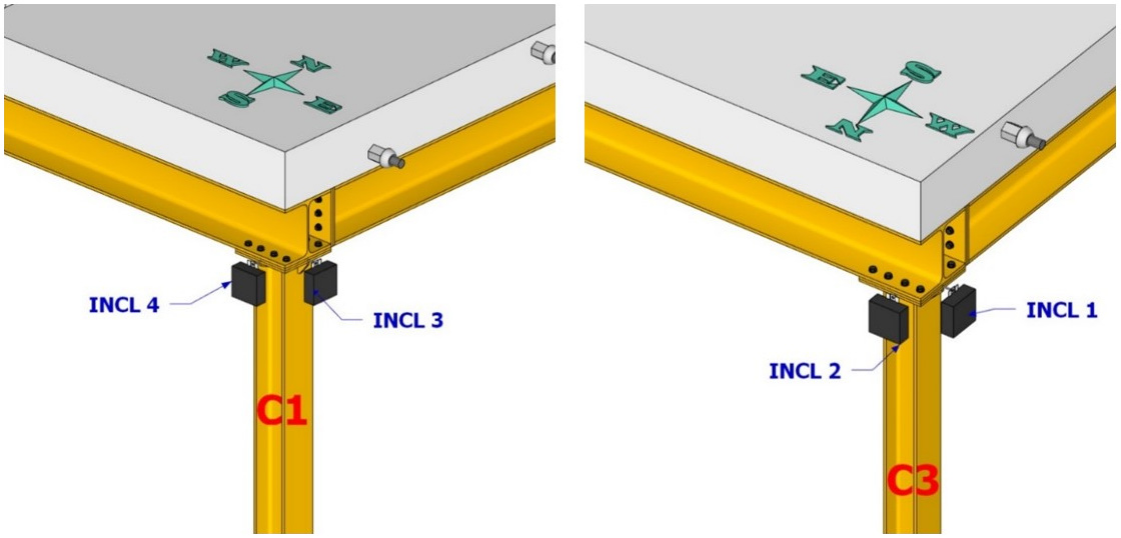
Position of inclinometers at top section of columns C1 and C3.

## Excitation

**Figure 11.**
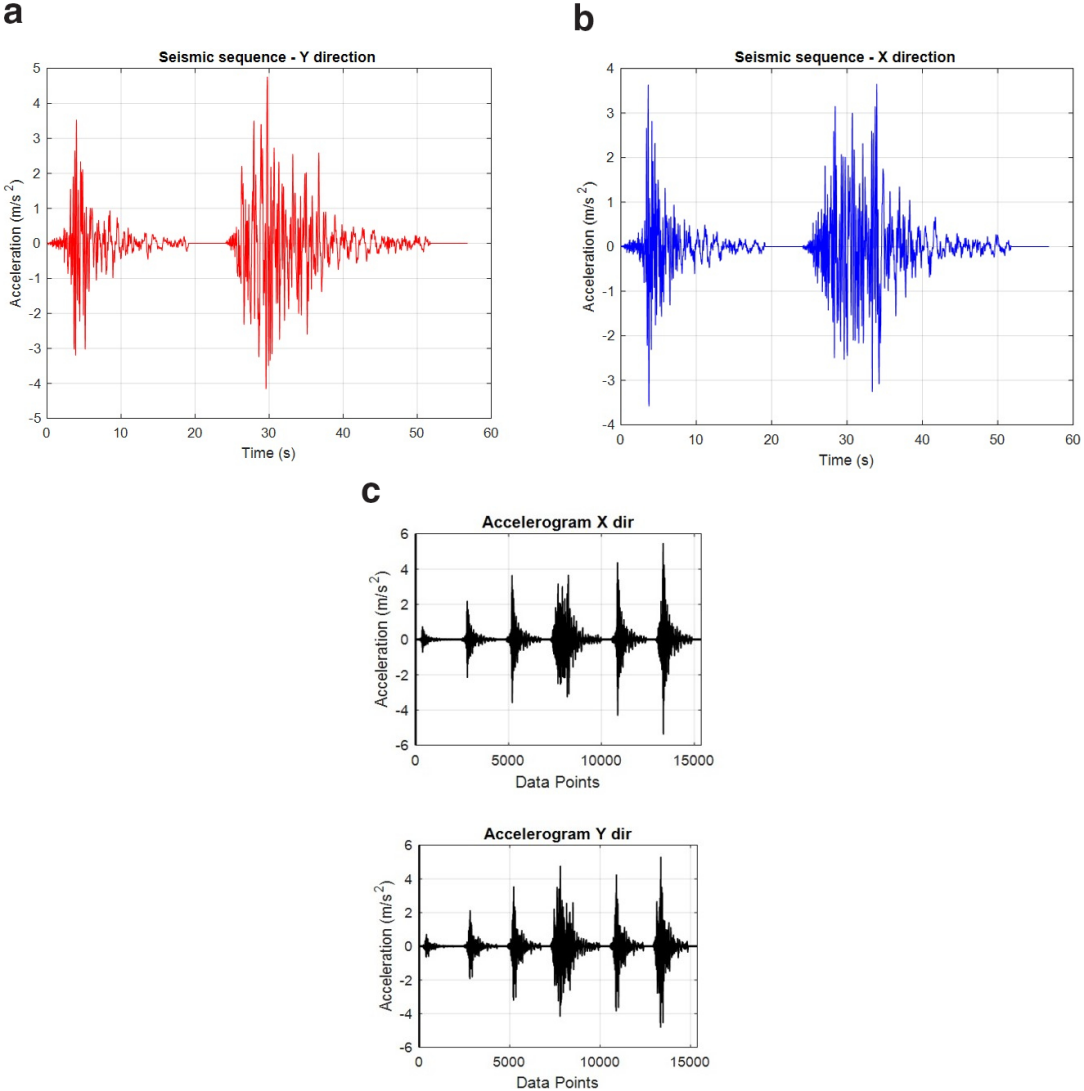
Seismic records for the: ( **a**) longitudinal, Y-direction, ( **b**) transverse, X-direction and, ( **c**) final sequence of records (X,Y) as applied.

As the scope of the tests includes the study of the effects of sequential seismic records occurring concurrently in orthogonal directions, a pair of sequentially applied natural earthquake signals were studied for being applied in the longitudinal and transverse direction of the structure, commencing and finishing concurrently ( Figure 11). Two ground motion records referring to the 2016 Central Italy earthquakes were selected for this purpose - the records were extracted from the Engineering Strong-Motion Database (ESM) ( Luzi *et al.,* 2020). Preliminary analyses considering different combinations regarding the specimen axis along which each one of the pairs is applied and the direction (sign) of each record in the combination, led to the conclusion that the most detrimental combination would be with record in Figure 11a been applied along the longitudinal (E-W) specimen (Y) axis, while record in Figure 11b being applied along N-S specimen (X) axis. Next, scaled versions (20%-, 60%-, 100%-, 120%- and 150%-scale) of these two "basic" records were considered of being applied sequentially, with the ensemble of acceleration time histories finally applied concurently along X and Y directions of the structure being depicted in Figure 11c.

## Testing program

The test matrix developed for the experimental campaign is summarised in Table 2 and in the same way was the dataset organised.

**Table 2.** Test matrix for the 3D steel frame specimen in the laboratory.

No.	Description	Note
**1**	Free vibration test: Y direction	10-mm pull-back
**2**	Free vibration test: X direction	10-mm pull-back
**2**	Quasi-static cyclic test: X direction	max. displacement: ± 27.5 mm
**4**	Quasi-static cyclic test: Y direction	max. displacement: ± 27.5 mm
**5**	Pseudo-dynamic test	two concurrent sequences of records applied

The specimen was first subjected to free vibration tests along both of its main axes to identify its modal properties. Subsequently, the specimen was subjected to quasi-static cyclic tests also along both of its main axes, which reached a maximum floor displacement of 27.5 mm in both directions. Finally, a series of pseudo-dynamic tests were performed on the specimen using the pre-selected ground motion records, considering incremental scaling factors for the ground motion intensity from 0.2 to 1.5.

## Free vibration test

**Figure 12.**
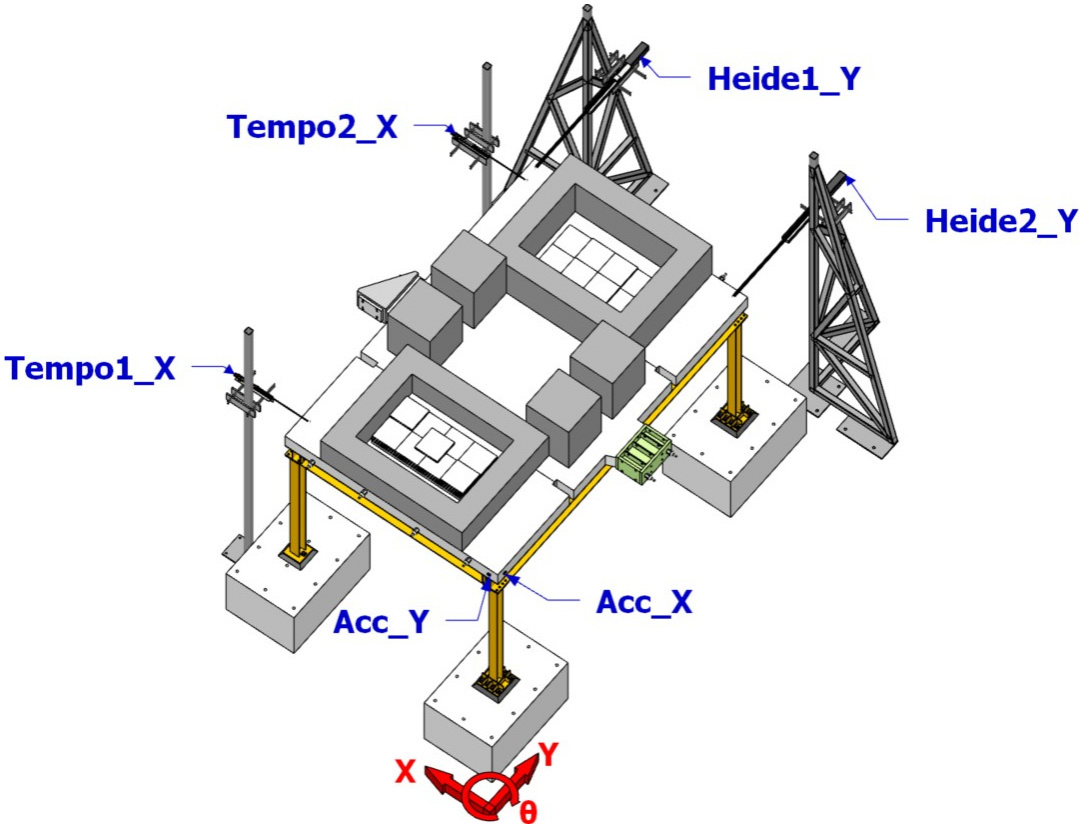
Free vibration testing layout.

The free vibration tests (layout demonstrated in Figure 12, "Tempo"-"Heide" denote optical displacement sensors and "Acc*_*" denote accelerometers) were performed along each of the main axis of the specimen, yielding the dynamic properties of the specimen per Table 3. The first global mode of the specimen was identified via free vibration in the X direction, which is a translational mode along the X direction with a period of 0.69 sec. The second and third global modes, associated with periods of 0.62 and 0.49 sec, are found to be rotational modes due to the asymmetry of the specimen in the Y direction caused by the different configurations of column base connections.

**Table 3.** Periods of vibration of the specimen.

Vibration direction	Mode 1	Mode 2	Mode 3
**Y (E-W)**	-	0.62 sec (rotational)	0.49 sec (rotational)
**X (N-S)**	0.69 sec (translational X)	-	-

## Photos and videos

The specimen response was monitored and recorded via cameras, both for what regards the overall vibration response of the specimen, as well for what concerns the response at the bases of the columns (which was also the target of the research).

Photographic documentation during the construction of the specimen and photos taken both during testing and following completion of the campaign, are shared with the dataset. Videos recorded during testing are also shared with the dataset.

## Test observations

**Figure 13.**
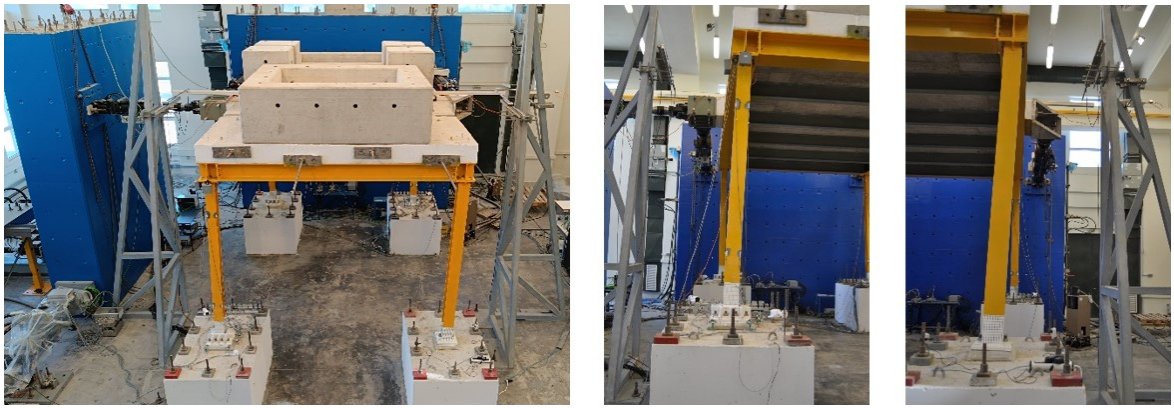
Deformed shape of the specimen.

**Figure 14.**
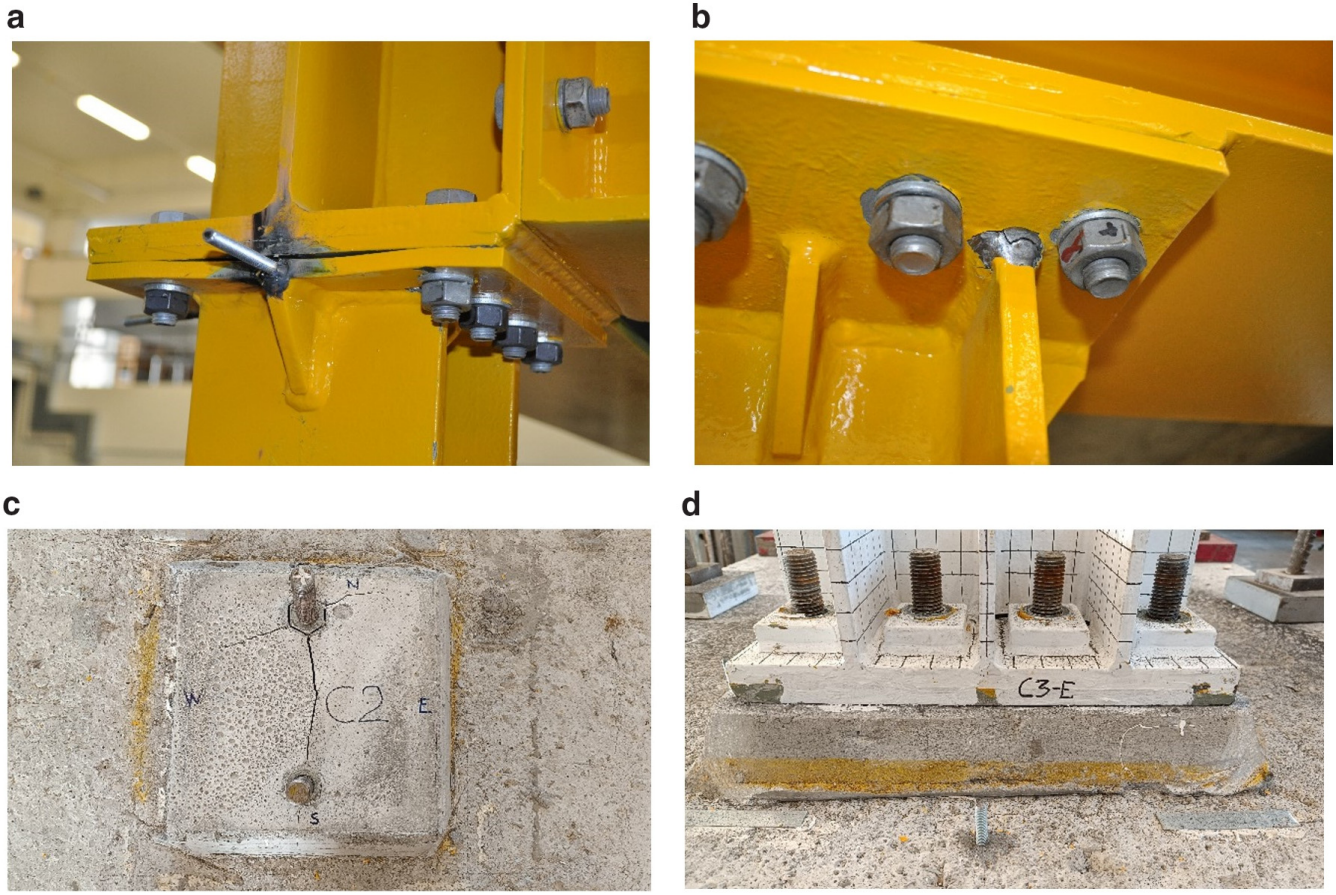
Examples of damage observed during the pseudo-dynamic tests: ( **a**) yielding of plates; ( **b**) fracture of welds; ( **c**) cracking of the mortar beneath the unstiffened column-base connection; ( **d**) limited damage at the stiffened column-base connection.

Excessive torsion of the specimen was observed during testing due to the different types of connections employed at the column bases (deformed shape of the structure shown in Figure 13). This resulted significant local damage at beam-to-column and column-base connections, such as fracture of welds and yielding of plates at the top of columns, plus cracking of the mortar beneath the column-base plates (see Figure 14 for examples of local damage).

## Ethics and consent

Ethical approval and consent were not required.

## Data Availability

The complete dataset produced by this experimental campaign is available on the public repository Zenodo - https://doi.org/10.5281/zenodo.10866777 ( Stathas *et al*., 2024). On the basis of the ERIES Transnational Access Agreement and the research team regarding data publication, the files will be made publicly available on September 30, 2024. Earlier access to the data may be provided to reviewers upon conrtacting ORE Editorial Team. The project dataset is structured in four folders, comprising the metadata regarding general information, the specimen and the campaign (geometry, materials, test setup, excitation signals applied in each experiment, etc.). The actually measured data (values) are provided as Excel files in separate folders (one per test performed), clearly designating at the first lines of each file the physical parameter represented in each column and the relevant units. Photos and videos are also provideed, stored in different (evidently named) folders. Data are available under the terms of the Creative Commons Attribution 4.0 International license (CC-BY 4.0)

## References

[ref-1] Borzouie J MacRae GA Chase JG : Experimental studies on cyclic performance of column base weak axis aligned asymmetric friction connection. *J Constr Steel Res.* 2015;112:252–262. 10.1016/j.jcsr.2015.05.007

[ref-2] Borzouie J MacRae GA Chase JG : Experimental studies on cyclic performance of column base strong axis–aligned asymmetric friction connections. *J Struct Eng.* 2016;142(1): 04015078. 10.1061/(ASCE)ST.1943-541X.0001327

[ref-3] CEN: EN 1993-1-8: 2005. Eurocode 3: design of steel structures. Part 1-8: design of joints, comité Européen de normalisation.Brussels,2005a. Reference Source

[ref-4] CEN: EN 1998-1: 2005. Eurocode 8: design of structures for earthquake resistance - Part 1: general rules, seismic actions and rules for buildings, comité Européen de normalisation.Brussels,2005b. Reference Source

[ref-5] Clifton C Bruneau M MacRae G : Steel structures damage from the Christchurch earthquake series of 2010 and 2011. *B New York Soci Earthq Eng.* 2011;44(4):297–318. Reference Source

[ref-6] Cloete R Roth CP : Column base connections under compression and biaxial moments: experimental and numerical investigations. *J Constr Steel Res.* 2021;184(11): 106834. 10.1016/j.jcsr.2021.106834

[ref-7] Di Sarno L Paolacci F Sextos AG : Seismic performance assessment of existing steel buildings: a case study. *Key Eng Mat.* 2018;763:1067–1076. 10.4028/www.scientific.net/KEM.763.1067

[ref-8] Di Sarno L Pecce MR Fabbrocino G : Inelastic response of composite steel and concrete base column connections. *J Constr Steel Res.* 2007;63(6):819–832. 10.1016/j.jcsr.2006.08.007

[ref-9] Fasaee MAK Banan MR Ghazizadeh S : Capacity of exposed column base connections subjected to uniaxial and biaxial bending moments. *J Constr Steel Res.* 2018;148:361–370. 10.1016/j.jcsr.2018.05.025

[ref-10] Grilli D Jones R Kanvinde A : Seismic performance of embedded column base connections subjected to axial and lateral loads. *J Struct Eng.* 2017;143(5): 04017010. 10.1061/(ASCE)ST.1943-541X.0001741

[ref-11] Gutiérrez-Urzúa LF Freddi F Di Sarno L : Comparative analysis of code-based approaches for seismic assessment of existing steel moment resisting frames. *J Constr Steel Res.* 2021;181: 106589. 10.1016/j.jcsr.2021.106589

[ref-13] Inamasu H Kanvinde AM Lignos DG : Seismic design of non-dissipative embedded column base connections. *J Constr Steel Res.* 2021;177: 106417. 10.1016/j.jcsr.2020.106417

[ref-12] Kanvinde AM Grilli DA Zareian F : Rotational stiffness of exposed column base connections: experiments and analytical models. *J Struct Eng.* 2012;138(5):549–560. 10.1061/(ASCE)ST.1943-541X.0000495

[ref-17] Latour M Piluso V Rizzano G : Rotational behaviour of column base plate connections: experimental analysis and modelling. *Eng Struct.* 2014;68:14–23. 10.1016/j.engstruct.2014.02.037

[ref-14] Latour M Rizzano G : Full strength design of column base connections accounting for random material variability. *Eng Struct.* 2013a;48:458–471. 10.1016/j.engstruct.2012.09.026

[ref-15] Latour M Rizzano G : A theoretical model for predicting the rotational capacity of steel base joints. *J Constr Steel Res.* 2013b;91:89–99. 10.1016/j.jcsr.2013.08.009

[ref-18] Lee DY Goel SC Stojadinovic B : Exposed column-base plate connections bending about weak axis: I. numerical parametric study. *Int J Steel Struct.* 2008a;8(1):11–27. Reference Source

[ref-19] Lee DY Goel SC Stojadinovic B : Exposed column-base plate connections bending about weak axis: II. experimental study. *Int J Steel Struct.* 2008b;8(1):29–41.

[ref-20] Luzi L Lanzano G Felicetta C : Engineering Strong Motion Database (ESM) (Version 2.0).Istituto Nazionale di Geofisica e Vulcanologia (INGV).2020. 10.13127/ESM.2

[ref-21] Mahin SA : Lessons from damage to steel buildings during the Northridge earthquake. *Eng Struct.* 1998;20(4–6):261–270. 10.1016/S0141-0296(97)00032-1

[ref-22] Okazaki T Lignos DG Midorikawa M : Damage to steel buildings observed after the 2011 Tohoku-Oki earthquake. *Earthquake Spectra.* 2013;29(1_suppl):219–243. 10.1193/1.4000124

[ref-23] Seco LDS Couchaux M Hjiaj M : Column base-plates under biaxial bending moment. *Eng Struct.* 2021;231: 111386. 10.1016/j.engstruct.2020.111386

[ref-24] Stathas N Di Sarno L Wu J : Biaxial seismic response of base-column connections in sub-standard steel buildings: dataset. 2024. 10.5281/zenodo.10866777 PMC11499733 39449777

[ref-25] You YC Lee D : Effect of anchors on the seismic performance of exposed column-base plate weak-axis connections. *J Build Eng.* 2020;32: 101803. 10.1016/j.jobe.2020.101803

[ref-26] Zhou F Suita K Matsumiya T : Tests on steel column bases with T-stub connections. *J Struct Constr Eng.* 2004;69(581):117–125. 10.3130/aijs.69.117_2

