# Supplementary material for: Biaxial seismic response of base-column connections in sub-standard steel buildings: dataset
Source: Open Res Eur. 2024 Nov 27;4:161. Originally published 2024 Aug 5. [Version 2] doi: 10.12688/openreseurope.17563.2 (PMC11499733; doi:10.12688/openreseurope.17563.2)
Supplement: Peer review history

# Reviewer report: Reviewer response for version 2

**DOI:** 10.21956/openreseurope.20519.r47556

**Published:** 2024-11-28

**Authors:** Roberto Nascimbene

**Affiliations:** 1 IUSS - University School for Advanced Studies, Pavia, Italy

Authors properly addressed all the reviewer's comments.

Are sufficient details of methods and materials provided to allow replication by others?

Yes

Is the rationale for creating the dataset(s) clearly described?

Yes

Are the datasets clearly presented in a useable and accessible format?

Yes

Are the protocols appropriate and is the work technically sound?

Yes

Reviewer Expertise:

My area of expertise is in the field of this research.

I confirm that I have read this submission and believe that I have an appropriate level of expertise to confirm that it is of an acceptable scientific standard.

# Reviewer report: Reviewer response for version 1

**DOI:** 10.21956/openreseurope.18979.r45006

**Published:** 2024-10-23

**Authors:** Aman Mwafy

**Affiliations:** 1 United Arab Emirates University, Al Ain, Abu Dhabi, United Arab Emirates

This manuscript outlines the methodology adopted in a comprehensive full-scale testing program for the seismic performance assessment of steel column connections with the foundation under bidirectional earthquake loading. The article is well-written and includes several well-prepared figures. It reflects the effort made in this detailed study, which made its test results available to researchers. A few optional suggestions are provided below to help improve the overall quality of this manuscript further. 

 - The abstract provides a brief introduction to the issue and its significance and describes the methodology. In addition to the last sentence highlighting the benefits of the experiments in model calibration and future parametric studies, it may be helpful for readers to emphasize that the study enriches the literature with a dataset produced by the comprehensive testing campaigns, which is available to researchers.

 - Plain language summary: “two orthogonal horizontal directions” or any other alternative can better describe bidirectional earthquake loading than “two transverse directions”.

 - Some figures can be improved further. It is suggested that the text size of Figure 1 be increased and the caption of Figure 2 be edited to better describe the contents in ‘a’ and ‘b’. The text related to Figure 2 may also need some revisions for clarity.

Are sufficient details of methods and materials provided to allow replication by others?

Yes

Is the rationale for creating the dataset(s) clearly described?

Yes

Are the datasets clearly presented in a useable and accessible format?

Yes

Are the protocols appropriate and is the work technically sound?

Yes

Reviewer Expertise:

Structural Earthquake Engineering

I confirm that I have read this submission and believe that I have an appropriate level of expertise to confirm that it is of an acceptable scientific standard.

# Response

**Published:** 2024-11-03

**Authors:** Bousias Efstathios

**Affiliations:** Civil Engineering, University of Patras, Patras, Peloponnisos Dytiki Ellada ke Ionio, Greece

The reviewer's suggestions were very constructive and are greatly appreciated. Indeed, all of them have been used in preparing a revision of the menuscript which will be uploaded soon.

# Reviewer report: Reviewer response for version 1

**DOI:** 10.21956/openreseurope.18979.r43069

**Published:** 2024-10-21

**Authors:** Shahab Ramhormozian

**Affiliations:** 1 Auckland University of Technology, Auckland, Auckland, New Zealand

This article is about an experimental research programme on the response of exposed column-base. Bi-directional Pseudo-Dynamic tests were undertaken, and two types of column-base plate connections namely stiffened and unstiffened were considered, representing the base connections of an external MRF, and an internal gravity frame respectively. The experiments included free vibration, quasi-static cyclic, and pseudo-dynamic tests.

 The article provides a clear background and rationale for the research as well as clear explanations and details of the experiments. The dataset is well organized and usable. I recommend this article to be accepted.

Are sufficient details of methods and materials provided to allow replication by others?

Yes

Is the rationale for creating the dataset(s) clearly described?

Yes

Are the datasets clearly presented in a useable and accessible format?

Yes

Are the protocols appropriate and is the work technically sound?

Yes

Reviewer Expertise:

Behaviour and design of steel structures - structural and earthquake engineering - low damage seismic resisting systems

I confirm that I have read this submission and believe that I have an appropriate level of expertise to confirm that it is of an acceptable scientific standard.

# Reviewer report: Reviewer response for version 1

**DOI:** 10.21956/openreseurope.18979.r44047

**Published:** 2024-09-27

**Authors:** Roberto Nascimbene

**Affiliations:** 1 IUSS - University School for Advanced Studies, Pavia, Italy

Engaging with such a paper as a reviewer has been truly enjoyable. The content showcases a strong structural framework, articulated presentation, and a research methodology that is both lucid and equitable. The topic is really less investigated by the research community even if it is so important for the consequences related to the catastrophic effects that can happen. Hence, I want to endorse its acceptance. However, I have some comments and suggestions for the authors. These insights are aimed at contributing to the continual improvement of the manuscript and elevating its overall quality.

 1. The findings, and procedure are well explained and well-written in the abstract. However, I have one suggestion: are all these acronyms truly necessary in the abstract? Specifically, acronyms like HITBASE, STRULAB, and ERIES could be omitted to improve readability. While these terms are important and can be used in the main body of the paper, the abstract should remain as accessible and clear as possible for a broad audience.

 2. Specifically, Figure 1 could be improved in terms of quality. Some of the text within the figure is difficult to read due to its small size. I suggest that the authors enhance the clarity of the figure by increasing the text size and improving the overall resolution to ensure that all elements are easily readable.

 3. When the authors state, 'Excessive torsion of the specimen was observed during testing,' it would be helpful if they could provide a quantification of this torsion. For example, they could include a percentage of torsional deformation, an angle of rotation, or any similar measurable parameter. This would give the reader a clearer understanding of the extent of the torsional effects observed and provide a more precise interpretation of the results.

Are sufficient details of methods and materials provided to allow replication by others?

Yes

Is the rationale for creating the dataset(s) clearly described?

Yes

Are the datasets clearly presented in a useable and accessible format?

Yes

Are the protocols appropriate and is the work technically sound?

Yes

Reviewer Expertise:

My area of expertise is in the field of this research.

I confirm that I have read this submission and believe that I have an appropriate level of expertise to confirm that it is of an acceptable scientific standard, however I have significant reservations, as outlined above.

# Response

**Published:** 2024-11-03

**Authors:** Bousias Efstathios

**Affiliations:** Civil Engineering, University of Patras, Patras, Peloponnisos Dytiki Ellada ke Ionio, Greece

The reviewer's insights and suggestions are greatly appreciated. Comments (1) and (2) have been considered in the revised version of the manuscript. Regarding comment (3): as the submitted manuscript is a "Data Note", its scope is to provide metadata only - indeed, in the data files uploaded to Zenodo (structured in the way explained in the manuscript) include measurements of center-of-mass of the specimen which can be used by interested users for further processing.
